# Immunostimulating and Anticancer Activities of the Pectic Polysaccharide from *Panax ginseng* Leaves Treated with High Pressure/Enzyme Process

**DOI:** 10.3390/cimb47040257

**Published:** 2025-04-07

**Authors:** Seung-U Son, Ki Rim Hong, Kwang-Soon Shin

**Affiliations:** 1 Precision Nutrition Research Group, Korea Food Research Institute, Wanju-gun 55365, Republic of Korea; suson@kfri.re.kr; 2 Department of Food Science and Biotechnology, Kyonggi University, Suwon 16227, Republic of Korea

**Keywords:** *Panax ginseng*, polysaccharide, pectin, immune system, lung cancer

## Abstract

This study was designed to investigate the immunostimulatory and anticancer efficacies of pectic polysaccharides from ginseng leaves treated using the high-pressure extraction method (HPEM). The isolation of polysaccharides using HPEM resulted in 1.35-fold higher polysaccharide yields than those obtained using the commonly used hot water extraction method. In addition, component sugar analysis of ginseng-leaf-derived polysaccharides (GLHP) showed the presence of nine different types of monosaccharides, including galacturonic acid, galactose, rhamnose, and arabinose, which are characteristic of pectic polysaccharides. In addition, GLHP effectively induced activation of the complement system, and macrophages stimulated with GLHP showed enhanced production of cytokines such as IL-6, IL-12, and TNF-α. Intravenous (i.v.) and oral administration (p.o.) of GLHP significantly increased the cancer-cell-killing ability of spleen-derived NK cells. In a lung-cancer-bearing mouse model using Colon26-M3.1 carcinoma, prophylactic i.v. and p.o. GLHP potently inhibited 95.2% and 33.5% of lung cancer, respectively. Furthermore, GLHP showed significant anticancer effects, even in mice with NK cell dysfunction, via the anti-asialo GM1 antibody. These effects may be related to the cancer-cell-killing effects of cytotoxic T lymphocytes (CTL). Therefore, GLHP, a polysaccharide isolated from ginseng leaves using HPEM, has a potent anticancer effect, and these effects are closely related to the stimulation of various immune factors.

## 1. Introduction

The immune system quickly and accurately removes several factors that adversely affect homeostasis, including foreign pathogens, bacteria, viruses, and cancer cells [1]. These systems maintain complex responses to various factors including the complement system, macrophages, natural killer (NK) cells, and T cells [2]. The complement system is an essential factor in the innate immune response and is activated by the recognition of various targets. To date, more than 30 complement proteins and regulators have been identified throughout the body that undergo a series of reactions and consequently form a membrane attack complex (MAC) on the target surface, and subsequently, the MAC penetrates the target surface and induces lysis. Several proteins formed during the complement activation process are known to initiate immune responses in various immune cells, including macrophages and dendritic cells [3]. Macrophages are representative effectors that perform various functions in the immune system. Briefly, activated macrophages elaborately regulate overall immune response by secreting various cytokines such as interleukin (IL)-4, IL-6, and IL-12 and tumor necrosis factor (TNF)-α. Cytokine secretion within the normal range has potent killing effects against the target by recruiting various immunocytes to nearby cancer cells and pathogens. In particular, IL-12 secreted from antigen-presenting cells such as dendritic cells and macrophages potently induces a Th1 type immune response, consequently enhancing cytotoxicity against NK cells and cytotoxic T lymphocytes (CTL) [4]. NK cells and CTL neutralize targets by secreting powerful cytolytic proteases such as perforin and granzyme B; however, there is a significant difference in the initiation of these killing effects [5,6]. CTL must be preemptively primed with dendritic cells, macrophages, and CD4^+^ T cells to remove the targets. CTL awaken only after a highly complex sensitization step and are converted into antigen-specific killers [7]. In contrast, NK cells skip this complicated process and recognize cells lacking major histocompatibility class (MHC) molecules I as ’abnormal’ or ’non-self’ and destroy their targets [8]. In many studies, these immune cells have been proven to have a remarkable target-killing effect, which allows us to expect anticancer effects from immunostimulation.

Various natural materials are being studied for immune system activation; however, plant-derived polysaccharides have received increasing attention due to their low toxicity and wide therapeutic spectrum [9]. The hot-water extraction method (HWEM) is traditionally used for the extraction of plant-derived bioactive polysaccharides; however, hydrothermal extraction results in a highly crude state. In general, separation by HWEM involves various substances such as pigments and proteins; therefore, subsequent separation and purification processes are more difficult [10]. In addition, this has the disadvantages of a long extraction time and low yield; therefore, various extraction methods, such as alkali, ultrasound, high-pressure, and microwave extraction, are being investigated to compensate for this. The high-pressure treatment of food materials was first devised in the early 1900s as a non-heat treatment method that can ensure food safety in a method similar to pasteurization, and meet consumer needs. In the early 2000s, high-pressure extraction method (HPEM) technology was first applied for the extraction of active substances and was found to result in higher yields and shorter processing times [11]. Since HPEM is conducted under airtight conditions, energy loss associated with solvent evaporation and phase transitions is minimized. In addition, HPEM requires lower temperatures for penetration of solvents and acceleration of polysaccharide diffusion. Accordingly, HPEM presents a more environmentally sustainable approach than conventional extraction techniques [12]. *Zizyphus jujube* polysaccharides extracted using HPEM exhibited greater prebiotic effects compared to those obtained through HWEM [13]. Additionally, large-leaf yellow tea polysaccharides extracted via HPEM demonstrated a more pronounced ameliorative effect on alcohol-induced liver injury [14].

The *Panax ginseng* (ginseng) extract has been proven to possess various biological activities. Many studies have shown that there is high physiological activity in its leaves, berries, and roots, which are mainly used in various products [15,16]. Ginseng leaves and berries are harvested every year; however, most of them are discarded, and studies applying HPEM to ginseng leaves are insufficient. Therefore, in this study, the active polysaccharide fraction was separated from ginseng leaves using HPEM, and its effect on immune activity was investigated. Furthermore, the achievement of these goals will contribute to new application methods and increase the added value of ginseng leaves.

## 2. Materials and Methods

### 2.1. Isolation of Polysaccharide Fractions from Panax ginseng Leaves

**Figure 1:**
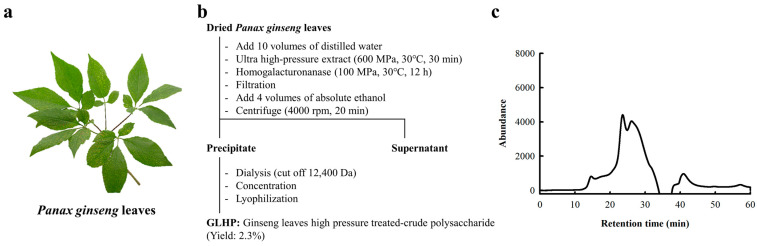
*Panax ginseng* leaves used for this study (**a**). Isolation scheme of GLHP isolated from *Panax ginseng* leaves (**b**) and elution patterns of GLHP (**c**). Elution patterns were obtained using high-performance liquid chromatography equipped with a Superdex G-75 packed column.

The ginseng leaves used in this study were purchased from a local market (Gyeongdong Market, Republic of Korea) (Figure 1a). Ginseng leaves harvested within 1–2 months of sowing were used in this study. The dried ginseng leaves were crushed and homogenized in a 10-fold (*v/w*) volume of distilled water. Following this, the suspension was reacted under 600 MPa and 30 °C conditions for 3 min, and the product was subsequently reacted with commercial pectinase (Novozyme, Bagsvaerd, Denmark) under 100 MPa and 30 °C conditions for 12 h. Residue and extract were separated via centrifuge (4000 rpm, 10 min, 4 °C), and polysaccharide fractions were precipitated through adding three times the volume of 99% ethanol. The precipitate was obtained through centrifuge (4000 rpm, 20 min, 4 °C), and re-dissolved using a small amount of distilled water. Subsequently, the resulting solution was dialyzed using a dialysis tubing bag (cut-off 12,400 Da; Sigma Aldrich, Saint Louis, MO, USA) for 3 days, lyophilized, and the dried powder was named ginseng leaf high-pressure/enzyme-treated crude polysaccharide (GLHP).

### 2.2. Experimental Animals

Specific pathogen-free (SPF) grade BALB/c (female, 6 weeks old) mice were used in all animal experiments. Mice were purchased from Saeron Bio (Seoul, Republic of Korea) and housed in a clean rack in an SPF room at Kyonggi University (Suwon, Republic of Korea). In the SPF room, the humidity and temperature were maintained at 55 ± 5% and 25 ± 1 °C, respectively. The lights were turned on/off every 12 h, and food and drinking water were ad libitum. All animal experiments were performed in accordance with the guidelines of the Institutional Animal Care and Use Committee of Kyonggi University (Approval No. 2017-008).

### 2.3. Chemical Properties Analysis

General chemical properties such as the neutral sugar [17], uronic acid [18], protein [19], and 2-keto-3-deoxy-D-manno-octonate (KDO) [20] contents of GLHP were analyzed according to previous reports. The components of the polysaccharide fraction were measured using a slightly modified 1-phenyl-3-methyl-5-pyrazolone (PMP) derivatization assay [21,22]. Briefly, a polysaccharide sample was hydrolyzed using 2 M trifluoroacetic acid (Sigma Aldrich) at 121 °C for 90 min and was marked with PMP under base conditions using 0.3 M NaOH (Sigma Aldrich) at 70 °C for 60 min. The resulting solution was neutralized using 0.3 M HCl (Sigma-Aldrich), and the PMP-marked monosaccharide fraction was selectively separated through an aqueous two-phase system using distilled water and chloroform (Junsei, Tokyo, Japan). Next, the solution was filtrated using a syringe filter (pore size: 0.45 μm; ADVANTEC, Dublin, CA, USA). The final product was analyzed using ultraviolet/visible detector (UVD)-high performance liquid chromatography (HPLC; Shimadzu, Kyoto, Japan) equipped with an Acclaim 120 C18 column (4.6 mm × 25 cm, 5 μm; Thermo Fisher Scientific, Sunnyvale, CA, USA). The analytical column was stabilized using a mixture of 0.1 M sodium phosphate buffer (pH 6.7) and acetonitrile in a ratio of 82:18 under an isocratic mobile phase assay.

The molecular weight (MW) of the polysaccharides was determined using a refractive index detector-HPLC (RID)-HPLC (Agilent, Palo Alto, CA, USA) equipped with a Superdex G-75 column (GE Healthcare, Piscataway, NJ, USA). The analytical column was stabilized using 0.05 M ammonium formate buffer (pH 5.5) under an isocratic mobile phase assay. A standard pullulan series (Showa Denko, Tokyo, Japan) was used to calculate the MW of the polysaccharide samples.

### 2.4. Complement System Activation

Complement system activation was determined using a method previously described by Mayer with slight modifications [23]. Blood samples were obtained from healthy adult volunteers at Kyonggi University (Suwon, South Korea). Whole blood was isolated using a centrifuge (13,000 rpm, 10 min, 4 °C), and the supernatant (normal human serum, NHS) was stored at −70 ℃ before use in the experiments. Next, gelatin-veronal buffer (GVB2+) (pH 7.4) was mixed with 0.5 mM Mg^2+^ and 0.15 mM Ca^2+^; subsequently, 50 μL of NHS, GVB2+, and each sample were mixed and kept in an ice water bath. After that, the mixture was incubated for 30 min at 37 °C, and 350 μL of GVB2+ was added. Sensitized red blood cell (IgM-sensitized sheep erythrocytes, EA cells, 1 × 10^8^ cells/mL) were added to the mixtures for performing a secondary reaction (4 °C, 60 min), and 2.5 mL of phosphate-buffered saline (PBS) was added to stop the reaction. The reactant was separated using a centrifuge for 10 min at 13,000 rpm and 4 °C, and the absorbance of the supernatant was measured at 412 nm using a spectrophotometer. Complement system activation was calculated as the inhibition rate (inhibition of 50% total complement hemolysis, ITCH_50_) in the negative control group, wherein only NHS, GVB^2+^, and distilled water were reacted.

### 2.5. Murine Peritoneal Macrophage Activation

In this study, 5% thioglycollate (Sigma Aldrich), which had matured for more than 3 months, was intraperitoneally injected to recruit murine peritoneal macrophages 4 days before macrophage harvesting. The induced macrophages were collected using PBS and seeded at a concentration of 2.5 × 10^5^ cells/well in a bottomed 96-micro well plate (SPL Life Science, Pocheon, Republic of Korea). Subsequently, various doses of GLHP were added to each well, and the media and lipopolysaccharide (LPS) were used as the negative control (NC) and positive control (PC), respectively. The plate was kept in a 37 °C and 5% CO_2_ incubator for 24 h; then, culture supernatant was collected for the measurement of the cytokine secretion level. The IL-6, IL-12, and TNF-α content of the culture supernatant was determined through enzyme-linked immunosorbent assay (ELISA). Mouse IL-6 and IL-12) ELISA kits were purchased from BD Bioscience (San Diego, CA, USA), and mouse TNF-α ELISA kit was purchased from Invitrogen (San Diego, CA, USA).

### 2.6. Splenic NK-Cell-Mediated Cytotoxicity Against Cancer Cell

The enhancing effects of the polysaccharides on splenic NK-cell-mediated cytotoxicity against cancer cells were investigated according to a previous study [24]. Doses such as 0.5 (GLHP-low dose; GLHP-L), 5 (GLHP-medium dose; GLHP-M), and 50 mg/kg (GLHP-high dose; GLHP-H) were intravenously injected twice, 1 day and 3 days before NK cell harvesting. The NK-cell-stimulating effects of the oral administration were confirmed by inoculating cancer cells after prophylactic administration of the samples once daily for 14 days. Boosted NK cells were harvested from the spleen using an NK cell isolation kit (Miltenyi Biotec, Bergisch Gladbach, Germany) according to the manufacturer’s recommendations. Cytotoxicity was determined by measuring the killing rate of NK cells (effectors) against YAC-1 lymphoma (target) cells, which are deficient in MHC class I expression. The mixture (ratio of effector/target (E/T); 50:1, 25:1, and 12.5:1) was seeded in round-bottomed 96-micro well plates (SPL life Science), and incubated in a 37 °C and 5% CO_2_ incubator for 24 h. Subsequently, lactate dehydrogenase (LDH) released from the target was measured using an EZ-LDH kit (DogenBio, Seoul, Republic of Korea).

### 2.7. Anticancer Metastasis Activities and NK-Cell Depleted Mice Model

The effects of polysaccharides on cancer metastasis were evaluated using Colon26-M3.1 carcinoma, a cancer cell line exhibiting high lung metastasis activity. Briefly, Colon26-M3.1 carcinoma (3.0 × 10^4^ cells/mouse) was inoculated intravenously (i.v.) to establish a lung cancer mouse model. Polysaccharide samples at various doses (0.5, 5, and 50 mg/kg) were administered via i.v. twice, 1 day and 3 days prior to cancer cell inoculation. The anticancer metastatic effects of oral administration were confirmed by inoculating cancer cells after prophylactic administration of the sample once daily for 14 days. Mice were sacrificed using a CO2 euthanasia chamber 14 days after cancer cell inoculation, and their lungs were extracted. Subsequently, the lungs were quickly washed with phosphate-buffered saline (PBS) and fixed using Bouin’s solution (Sigma-Aldrich), and the number of tumor colonies was counted using a microscope.

To confirm the NK-cell-dependent anticancer metastatic effects of GLHP, NK-cell-deficient mice were established using an anti-asialo GM1 antibody 2 days prior to cancer cell inoculation.

### 2.8. Splenic Cytotoxic T Lymphocyte-Mediated Cytotoxicity Against Cancer Cells

The enhancing effects of the polysaccharides on splenic CTL-mediated cytotoxicity against cancer cells were investigated according to a previous study [25]. Briefly, Colon26-M3.1 carcinoma (3.0 × 10^4^ cells/mouse) was inoculated for presensitization of CTL [26]. In addition, various doses of GLHP (0.5, 5, and 50 mg/kg) were prophylactically administered via i.v. twice, 1 day and 3 days before cancer cell inoculation. After 14 days, the boosted CTL were harvested from the spleen using a CD8^+^ T cell isolation kit (Miltenyi Biotec) according to the manufacturer’s recommendations. Cytotoxicity was determined by measuring the killing rate of CTL (effector) against Colon26-M3.1 (target) used in presensitization. The mixture (ratio of effector/target; 50:1, 25:1, and 12.5:1) was seeded in a round-bottomed 96-micro well plate (SPL life Science), and incubated in a 37 °C and 5% CO_2_ incubator for 24 h. Subsequently, the LDH released from the target was measured using an EZ-LDH kit (DogenBio).

### 2.9. Statistical Analysis

All experimental data are presented as mean ± standard deviation of the mean for experiments performed in triplicate, with different letters signifying statistical significance. Statistical significance was evaluated using a one-way analysis of variance and Duncan’s test for multiple comparisons. In all the experiments, a *p*-value < 0.05 was used as the threshold for statistical significance. Statistical analyses were conducted using IBM SPSS statistics 27.0 version (IBM Corp., Armonk, NY, USA).

## 3. Results

### 3.1. Preparation and Chemical Characteristics of Active Polysaccharide from Ginseng Leaves

The polysaccharides present in plant cell walls can be classified into starch, cellulose, hemicellulose, and pectic substances. Among them, pectin has been the target of several studies due to its potent therapeutic activity against various diseases such as hyperlipidemia, cancer, inflammatory bowel disease, and diabetes [27]. In addition, pectin can be extracted relatively simply because it exists in a free and flexible form between cellulose and hemicellulose [28]. In general, pectin is classified into homopolysaccharides and heteropolysaccharides. The homopolysaccharide region is a polymer in which galacturonic acid is straight-stretched with a α-1,4 glycosidic bond, and is expressed as homogalacutornan (HG) and does not show any specific biological activity. In contrast, the heteropolysaccharide region is composed of various monosaccharides, such as arabinose (Ara), galactose (Gal), rhamnose (Rha), and galacturonic acid (GalA), connected by complex glycosidic bonds and represented by rhamnogalacturonan (RG)-I or -II. In addition, the biological activities of pectin-derived polysaccharides originate mostly from RG. Therefore, we isolated RG-type polysaccharides from ginseng leaves without the HG region.

GLHP was obtained by HPEM, enzymatic hydrolysis, and ethanol precipitation, as described in Section 2.1. This process was intended to extract a high yield of polysaccharides in a short time under high-pressure conditions, and remove the HG region present in pectin using homogalacturonanase. The results (Figure 1b) confirmed that GLHP had a yield of 2.3% compared to the dried ginseng leaves used. On the other hand, we performed other isolations using HWEM for comparison with HPEM, and the yield was only 1.7%. Therefore, it was confirmed that polysaccharide extraction using HPEM had a yield that was approximately 1.35 folds higher than that obtained using HWEM. The elution patterns of GLHP obtained by size exclusion chromatography (SEC) suggested that GLHP existed in a crude state (Figure 1c), and these results strongly suggest the possibility that HG was not completely decomposed, as well as the presence of RG-I or RG-II as a mixture. Furthermore, the chemical properties (Table 1) indicated that GLHP was composed of more than 90% sugars, including neutral sugars (53.9 µg/100 µg of GLHP), uronic acid (26.3 µg/100 µg of GLHP), and KDO (2.3 µg/100 µg of GLHP). KDO is a rare sugar found in RG-II, which supports the possibility that RG-II is present in GLHP. The results of the sugar composition analysis (Table 1) were mostly consistent with the monosaccharides of RG-type polysaccharides, such as GalA, Rha, Ara, Gal, xylose (Xyl), and fucose (Fuc). Consequently, it was confirmed that the GLHP isolated using HPEM were a mixture of typical RG-type polysaccharides, which were considered to contain both RG-I and RG-II. For simple sample preparation, we conducted an experiment on immune activity using GLHP without an additional purification process.

### 3.2. Complement System Activation by GLHP Isolated from Ginseng Leaves

**Figure 2:**
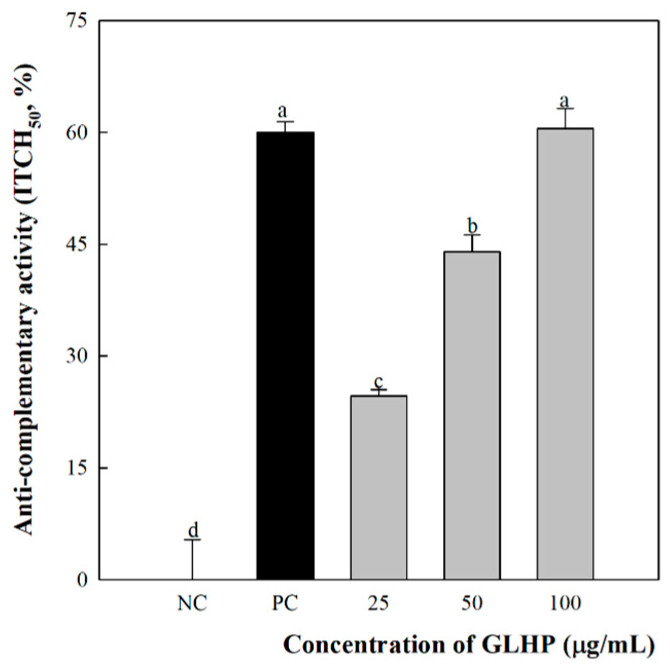
Anticomplementary activity of GLHP isolated from *Panax ginseng* leaves. NC (distilled water) and PC (PSK, 500 μg/mL) represent the negative and positive controls, respectively. All data are presented as the mean ± standard deviation (n = 3); the different letters (a–d) indicate statistical significance (*p* < 0.05). NC and PC represent the negative and positive controls, respectively. PSK, polysaccharide- K. PSK is an immunostimulating polysaccharide isolated from *Coriolus versicolor* that was used as a PC.

The activation of the complement system is carefully controlled by several cascade steps, and various complement component proteins play multiple roles against other immunocytes and modulatory systems. Consequently, activation of the complement system not only directly protects the body by quickly removing the target, but also provides potent stimulatory effects across the immune system. These inhibitory functions also include persistent targets, such as cancer [29]. In addition, C3b produced during complement system activation binds to complement receptor-1, -2, and -3 expressed on the cell membrane surface of macrophages to promote phagocytosis and cytokine secretion. Therefore, the effect of GLHP on the innate immune system was investigated by examining complement system activity. As a result (Figure 2), polysaccharide-K (PSK; Krestin), a commercial immuno-enhancing anticancer drug used in the PC group, induced a hemolytic effect of 60% against sheep red blood cells. In contrast, the NC group, treated with distilled water alone, did not show any hemolytic effects. However, in the experimental group treated with GLHP, it was confirmed that the hemolytic effect increased in a dose-dependent manner (25, 50, and 100 μg/mL; 24.6%, 44%, and 60.5%, respectively). Moreover, these effects were similar to those of PSK used as PC at a concentration of 100 μg/mL. Antigen-presenting cells such as macrophages not only produce proteins necessary for complement activation but also express complement receptors such as C3a and C5a receptors. Many studies have shown that these receptors are essential in the process of maturation, differentiation, and antigen presenting [30,31]. Therefore, the effects of GLHP on the complement system suggest the possibility of cascading macrophage activation.

### 3.3. Cytokine Secretion of Macrophages by GLHP Isolated from Ginseng Leaves

Macrophages guard all tissues in the body and can also be called microglia or Kupffer cells, depending on where they reside [32]. In dendritic cells, they play various critical roles in protecting the body by inducing acute inflammatory reactions at the forefront of defense. For example, various cytokines secreted by macrophages are multifunctional proteins that enhance the killing abilities of effector cells and lymphocytes [33]. In addition, macrophages activated by cytokines not only rapidly remove foreign pathogens through phagocytosis but are also deeply involved in adaptive immunity by presenting a peptide residue remaining after destruction of the target. Therefore, measurement of cytokine secretion by macrophages is considered an important indicator for evaluating the degree of immunostimulatory activity.

**Figure 3:**
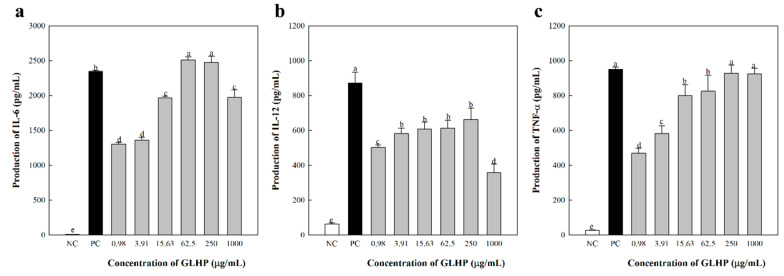
Effects of GLHP isolated from *Panax ginseng* leaves on IL-6 (**a**), IL-12 (**b**), and TNF-α (**c**) secretion from murine peritoneal macrophages in vitro. Murine peritoneal macrophages (2.5 × 10^5^ cells/well) were treated with various concentrations of polysaccharides for 24 h. NC (medium) and PC (LPS, 1 μg/mL) represent the negative and positive controls, respectively. All data are presented as the mean ± standard deviation (n = 3); the different letters (a–e) indicate statistical significance (*p* < 0.05). LPS, lipopolysaccharide.

As shown in Figure 3, the NC group treated only with medium showed very weak cytokine secretion, whereas the PC group treated with LPS showed highly explosive cytokine secretion. In addition, supply of GLHP to macrophages dose-dependently increased the secretion of IL-6 (Figure 3a), IL-12 (Figure 3b), and TNF-α (Figure 3c). This tendency was seen up to a concentration of 250 μg/mL, and the secretion of IL-6 and IL-12 was slightly reduced in the experimental group at the highest treatment concentration of 1000 μg/mL. Furthermore, IL-6, IL-12, and TNF-α all had the highest statistical values at concentrations of 250 μg/mL, and IL-6 and TNF-α showed similar values to the LPS used as PC at concentrations of 250 μg/mL. The decrease in the secretion of Th1 type cytokines such as IL-12, in high-dose samples, is similar to that reported by Lv et al. [34] and Lin et al. [35]. These results are closely related to the ability of macrophages to regulate the Th1/Th2 type immune response balance [27]. In particular, IL-12 is a cytokine that activates the killing ability of effectors such as NK cells and CTL; therefore, it is known to be an important factor in predicting the anticancer effects of active substances. Among them, NK cells are powerful lymphocytes that drive the initial immune response, along with macrophages and the complement system. Our additional aim was to confirm the activation of NK cells by GLHP.

### 3.4. Cancer Cell Killing Effects Through NK Cell Stimulated by GLHP Isolated from Ginseng Leaves

NK cells are lymphocytes belonging to the innate immune system with strong cytolytic capabilities, accounting for 2–7% of peripheral blood in mice and 5–15% in humans. Recently, they have been in the spotlight as important factors in anticancer treatment due to their potent killing ability. Briefly, several stressed cells, such as virus-infected cells, senescent cells, and cancer cells lose the expression of MHC class I molecules [25,36]. NK cells accurately recognize this abnormal situation and secrete potent cytolytic proteases, such as perforin and granzyme B, to neutralize their target [37,38]. Previous studies have confirmed that the ginseng-leaf-derived polysaccharide GLHP activates the complement system and macrophages belonging to the innate immune system. Therefore, we investigated the effects of GLHP on NK cells, which are the most potent effectors of the innate immune system.

**Figure 4:**
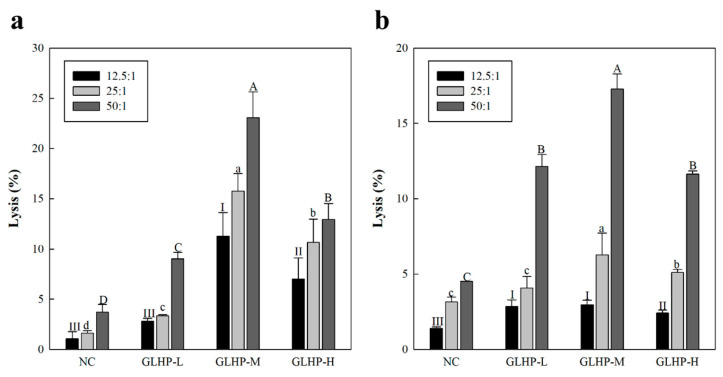
Effects of intravenous (**a**) and oral (**b**) administration of GLHP isolated from *Panax ginseng* leaves on the ex vivo cytolytic activity of NK cells. GLHP-administered NK cells were co-cultured with the YAC-1 lymphoma in a CO_2_ incubator (37 °C) for 6 h. NC (saline) represents the negative control. All data are presented as the mean ± standard deviation (n = 3); the different letters (I–III, a–d, A–D) indicate statistical significance (*p* < 0.05). NC represents the negative controls.

As a result (Figure 4a), i.v. administration of GLHP enhanced the killing ability of NK cells against YAC-1 lymphoma. In the NC group administered with PBS only, approximately 3.7% of the cancer cells were lysed when the E/T ratio was set to 50:1. However, GLHP administered groups, such as GLHP-L and GLHP-M (9.0% and 23.1%, respectively), showed an increase in cancer-cell-killing effects in a dose-dependent manner. These tendencies were slightly reduced and reversed in the GLHP group (12.9%), similar to previous macrophage activation results. Moreover, oral administration of GLHP enhanced NK-cell-mediated cytotoxicity against YAC-1 lymphoma cells (Figure 4b). Since many studies have shown that NK cells have strong anticancer capabilities, our results suggest that stimulation with GLHP may lead to superior anticancer effects.

**Figure 5:**
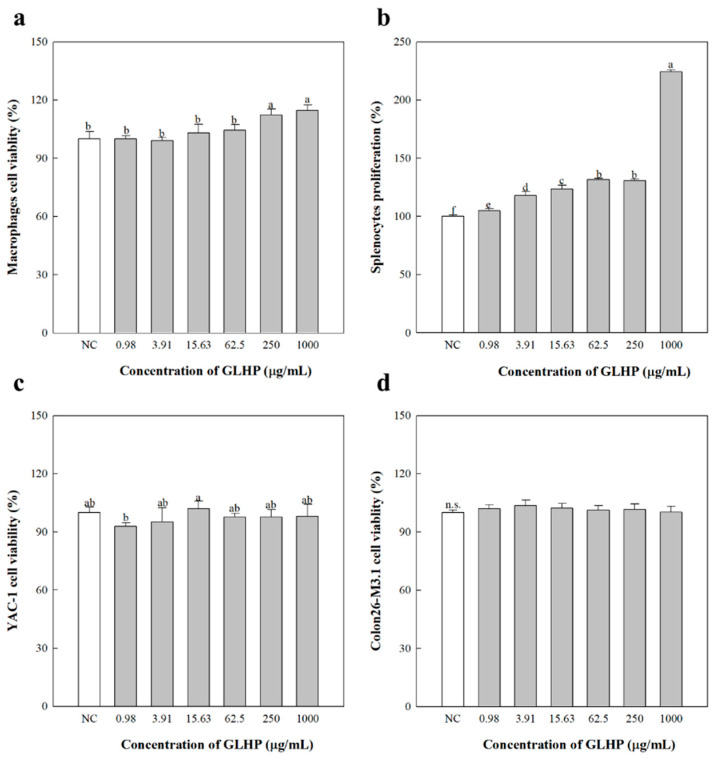
Effects of GLHP isolated from *Panax ginseng* leaves on the cell viability of primary cells and cancer cell lines in vitro. Primary cells used were peritoneal macrophages (**a**) and total splenocytes (**b**), and the cell lines used were YAC-1 lymphoma (**c**) and Colon26-M3.1 carcinoma (**d**). All cells (2.5 × 10^5^ cells/well) were treated with various concentrations of polysaccharides for 24 h. NC (medium) represent the negative controls. All data are presented as the mean ± standard deviation (n = 3); the different letters (a–f) indicate statistical significance (*p* < 0.05). LPS, lipopolysaccharide. n.s., no significant difference at *p* < 0.05.

Before conducting experiments on the anticancer effects of GLHP, the direct cytotoxicity of GLHP on normal and cancer cells was confirmed. GLHP did not show any cytotoxicity toward peritoneal macrophages or splenocytes isolated from mice (Figure 5). However, no proliferation or cytotoxicity effects were observed for YAC-1 lymphoma (c) and Colon26-M3.1 carcinoma (d). Therefore, it was considered that GLHP had no direct anticancer effects, and additional experiments on the inhibitory effects of GLHP in a cancer mouse model were performed.

### 3.5. Anticancer Effects of i.v. and Oral Administration of GLHP Isolated from Ginseng Leaves

**Figure 6:**
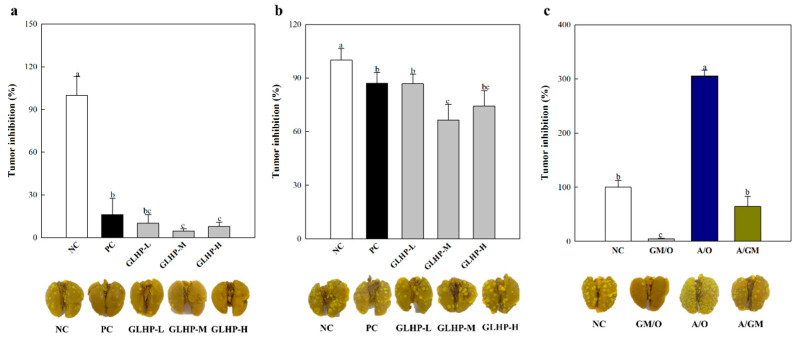
Inhibitory effects of intravenous (**a**) and oral (**b**) administration of GLHP on Colon26-M3.1 carcinoma-induced lung cancer bearing mice model. Effect of NK cell depletion on the anticancer activity of 5 mg/kg GLHP (**c**). BALB/c mice were intravenously or orally administered with GLHP at various doses and then intravenously inoculated with Colon26- M3.1 carcinoma. Rabbit anti-asialo GM1 serum was intravenously injected into the mice two days before inoculation with Colon26-M3.1 carcinoma cells to deplete NK cells. All data are presented as the mean ± standard deviation (n = 8); the different letters (a–c) indicate statistical significance (*p* < 0.05). NC (saline) and PC (PSK, 1000 μg/mL) represent the negative and positive controls, respectively. PSK, polysaccharide- K. PSK is an immunostimulating polysaccharide isolated from *Coriolus versicolor* that was used as a PC.

Previous results have confirmed that GLHP stimulates various immune factors, such as the complement system, macrophages, and NK cells. These were all found to be closely related to anticancer effects; therefore, we aimed to confirm the anticancer effects of GLHP in vivo. The anticancer effects of GLHP were verified using lung-cancer-bearing mice induced by Colon26-M3.1, which has a high ability to metastasize to the lung. First, i.v. administration was confirmed to have a dose-dependent cancer inhibitory effect on lung cancer compared to the NC group administered only PBS (Figure 6a). However, at the highest concentration, 50 mg/kg (GLHP-H 92.1%), a slight decrease in efficacy was observed compared with that at 5 mg/kg (GLHP-M; 95.2%). Moreover, these effects were superior to that of PSK used as the PC group (83.7%) even at the lowest experimental dose of 0.5 mg/kg (GLHP-L; 89.8%). From the above results, i.v. administration of GLHP was considered to have potent anticancer effects at all tested doses, and we aimed to further verify this effect through oral administration.

As shown in Figure 6b, the anticancer effect of oral administration was significantly reduced compared to i.v. administration at the same doses. However, all experimental groups (13.2%, 33.5%, and 25.7%, respectively) showed higher anticancer effects than PSK used as PC group (12.9%). In summary, our previous results confirmed that GLHP activates immune cells that inhibit cancer cells. In fact, the administration of GLHP showed potent anticancer efficacy in Colon26-M3.1-induced lung-cancer-bearing mice, and these effects were seen after both i.v. and oral administration. In addition, GLHP was most effective at a concentration of 5 mg/kg, both i.v. and orally. Therefore, to determine whether this anticancer effect is related to immune activity, we blocked the function of NK cells, the most effective tumor killer, using an anti-asialo GM1 antibody and then re-evaluated the anticancer effect. As shown in Figure 6c, the A/O (asialo GM1 only) group, in which NK cell function was suppressed, showed an explosive increase of approximately 3-fold in tumors compared to the NC group. However, the A/GM (asialo GM1/ GLHP-M combination) group was found to have significantly fewer cancer cells than the A/O group, although the function of NK cells was inhibited by asialo GM1. These effects were weaker than those of GM/O (GLHP-M only).

**Figure 7:**
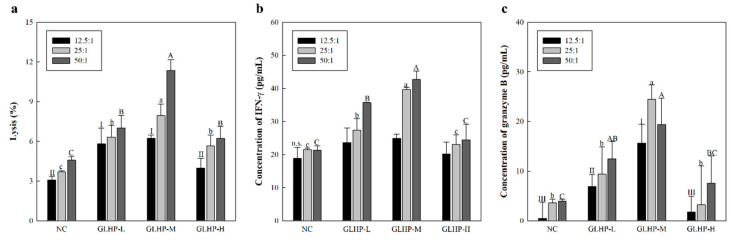
Effects of intravenous administration of GLHP isolated from *Panax ginseng* leaves on the cytolytic activity of CTL (**a**) and secretion of cytokines such as IFN-γ (**b**) and granzyme B (**c**). The Colon26-M3.1 carcinoma (3.0 × 10^4^ cells/mouse) was inoculated for presensitization of CTL. GLHP-administered CTL cells were co-cultured with the Colon26-M3.1 carcinoma in a CO_2_ incubator (37 °C) for 6 h. NC (saline) represents the negative control. All data are presented as the mean ± standard deviation (n = 3); the different letters (I–III, a–c, A–C) indicate statistical significance (*p* < 0.05). NC represents the negative controls. n.s.: not significant.

The above results (Figure 6c) not only suggest that the anticancer efficacy of GLHP is strongly related to the activation of NK cells, but also suggest that other immune factors act to show anticancer efficacy. Although there are various candidates for other immune factors, CTLs, such as NK cells, are known to possess a potent cancer-killing capacity. CTL is also known to enhance aggression against targets by secreting proteins such as grangyme B and interferon (IFN)-γ. Meanwhile, as CTL require presensitization, unlike NK cells, Colon26-M3.1 was inoculated to perform presensitization for CTL after administering GLHP. This study aimed to investigate the effects of GLHP by confirming the cytotoxicity of presensitized CTL against target cells after a sufficient sensitization period. Interestingly, as expected (Figure 6c), GLHP increased the cytotoxic capacity of CTL against Colon26-M3.1 (Figure 7a). Furthermore, granzyme B (Figure 7b) and IFN-γ (Figure 7c) secreted in the co-culture supernatant were observed to increase with GLHP administration. The activation of NK cells and CTL increases the possibility of positive contributions to systemic immunity through complex networks, and not just the destruction of cancer cells. Our future research goals will be to demonstrate and understand the molecular mechanisms of these complex immune networks.

## 4. Conclusions

In this study, we conducted HPEM and enzyme treatments to achieve a high yield of polysaccharides from ginseng leaves. As a result, a pectic polysaccharide (GLHP) was obtained in a relatively high yield, and the SEC elution pattern, chemical properties, and sugar composition results suggested that GLHP was a typical pectic polysaccharide. In addition, GLHP has been observed to enhance various immune factors, such as the complement system, macrophages, NK cells, and CTL. Additionally, a lung-cancer-bearing mouse model confirmed that the potent anticancer effect of GLHP was closely related to immune factors. The results presented in this study suggest that ginseng-leaf-derived pectic polysaccharides may serve as novel anticancer drugs. Immune responses occurring in the body or within a single cell through active polysaccharides are known to occur through a cascade of more complex signaling pathways than those suggested in this study. Nevertheless, the findings of the present study are limited in identifying the underlying mechanisms through molecular approaches. The detailed mechanisms responsible for the anticancer effects of GLHP may be elucidated through further investigation. Moreover, activation of the immune response is closely related to the sugar chain linkages of the active polysaccharide. Therefore, our goal was to reveal the correlation between the intracellular molecular mechanisms induced by GLHP and sugar chain linkages.

## Figures and Tables

**Table 1:** Chemical properties and sugar composition of GLHP isolated from *Panax ginseng* leaves.

Chemical Properties	GLHP (µg/100 µg)
Neutral sugar	53.9 ± 0.8
Uronic acid	26.3 ± 0.4
Protein	9.1 ± 0.7
KDO *-liked material	2.3 ± 0.0
**Sugar Composition**	**GLHP (µg/100 µg)**
Rhamnose	7.5 ± 0.3
Mannose	7.3 ± 0.0
Glucose	16.1 ± 0.4
Galactose	19.0 ± 0.6
Fucose	2.7 ± 0.5
Xylose	1.5 ± 0.3
Arabinose	12.5 ± 0.1
Glucuronic acid	5.3 ± 0.3
Galacturonic acid	10.1 ± 0.1

* **KDO**: 2-keto-3-deoxy-D-manno-octonate.

## Data Availability

Data will be made available on request.

## Abbreviations

The following abbreviations are used in this manuscript:

Ara	Arabinose
A/GM	Asialo GM1/GLHP-M combination
A/O	Asialo GM1 only
CTL	Cytotoxic T lymphocyte
E/T	Effector/target
Fuc	Fucose
Gal	Galactose
GalA	Galacturonic acid
GLHP	Ginseng leaves high pressure- enzyme polysaccharide
GLHP-L	GLHP-low dose
GLHP-M	GLHP-medium dose
GLHP-H	GLHP-high dose
GM/O	GLHP-M only
GVB	Gelatin-veronal buffer
HG	Homogalacturonan
HPEM	High-pressure extraction method
HWEM	Hot water extraction method
IL	Interleukin
ITCH_50_	Inhibition of 50% total complement hemolysis
i.v.	Intravenous
KDO	2-keto-3-deoxy-D-manno-octonate
LDH	Lactate dehydrogenase
LPS	Lipopolysaccharide
MAC	Membrane attack complex
MHC	Major histocompatibility complex
MW	Molecular weight
NC	Negative control
NHS	Normal human serum
NK	Natural killer
PMP	1-phenyl-3-methyl-5-pyrazolone
PC	Positive control
PSK	Polysaccharide-K
Rha	Rhamnose
RG	Rhamnogalacturonan
RID	Refractive index detector
TNF	Tumor necrosis factor
UVD	Ultraviolet visible detector
Xyl	Xylose
